# Dentition*Read before the Tennessee State Dental Association, June 7, 1913.

**Published:** 1913-08-15

**Authors:** B. B. O’Bannon

**Affiliations:** Memphis, Tenn.


### DENTITION.*

BY B. B. O'BANNON, D.D.S., MEMPHIS, TENN.

The fact that a considerable portion of the human family die before they have reached the period at which the last of the deciduous teeth should have erupted, and that the time of the greatest mortality is during the period in which the first teeth make their appearance, has led to the popular belief that the one is necessarily dependant upon the other, and that dentition is the almost exclusive cause of the high death rate of children, instead of more frequently being merely coincidental. That it is possible for a retarded or disturbed dental development to induce a very serious derangement must be admitted, but that it is the principle factor in inducing the great number of deaths that occur in children from digestive disorders can scarcely be maintained. There are many reasons for the contrary belief while there is nothing save the mere fact of coincidence to sustain the theory so commonly accepted without conclusive inquiry or adequate consideration. There is a lack of comprehension as to the true character of the disease that causes the high death rate in children. Digestive derangements are not the main factor if we except nervous disorders, as these are the only ones that can with propriety be urged as the probable cause of disturbances in dentition. Statistical summaries no where give the cutting of teeth as the cause of death Although the reflex disturbances due to d sordered dent t on undoubtedly in some instances are the prox mate, if not the direct cause of death, it is possible always to dist nguish them from other reflex disturbances. In any case, statistics should not pretend to present more than the immediate cause. But the fatal lesion being determined, it is comparatively easy to discover whether reflexes may readily induce it and from these probabilities determine their influence in

*Read before the Tennessee State Dental Association, June 7, 1913.

any given table. For instance, no one will urge that they can play any important part in any of the Zymotic disturbances which are chiefly chargeable with the majority of infantile deaths; while in affections of the brain, heart or kidneys they might be frequently responsible.

If it be granted that brain trouble may be due to reflex irritation, it is by no means established that the source lies in the teeth.

It has been observed that an infant passes through periods of growth and rest in the development of its body, the rest period being used by nature for the perfecting and maturing of that which has been added to the body of the infant by the period of growth. It has also been noted that children after a period of seeming suspension of development may within a few months add an inch or more to their statue, this is succeeded by a period of rest when the tissues pass through a process of maturing. It is well known that during these terms of rapid growth young persons are more liable to injuries and illness of different kinds than they are either before or after them. It should be remembered that the teeth like the other organs of the body have their distinct eras and that they develop with the rest of the body and not independently of it. When the child is erupting its first teeth it is at the same time developing a stomach and new digestive organs.

The process of teething, eruption or dentition, compromises that series of vital operations which cause the teeth to leave their crypts in the maxilla where the germs that form the teeth lay to pierce the gums and to take their places in the dental arches. Physiological dentition is divided into—First, dentition, or the eruption of the deciduous teeth; and Second, dentition, or the eruption of the permanent teeth.

### CAUSES OF ERUPTION.

It is evident that there are forces in the system that bring about the elevation of a tooth crown from its bed in the crypt to its position in the mouth, causing in its advent the absorption of the overlying structures. The crown elevation is due largely to the lengthening of the root end of the tooth, as root tissue is formed by the pulp the tooth is gradually pushed up and more root is formed and the process goes on until completed. Of course, the nutritional processes of resorption and rebuilding and blood pressure in the adjacent tissues must be taken into consideration.

### SYMPTOMS OF ERUPTION.

As a general rule the eruption of the deciduous teeth may be said to begin about the seventh month after birth, but of course the time varies. In ordinary first dentition slight local disturbances are so common as to be accepted as physiological. The resorption of the soft tissues around the tip of the crown of the tooth implies a condition of mild non-septic inflammation at that point. In more marked cases there is evidence of some local irritation, manifested by the behavior of the infant, and upon examination the gum is found to be somewhat redder in color than normal, and the temperature is elevated. Relief is produced by rubbing which reduces the hyperemia and the child is pleased to have its gums rubbed or to bite upon some hard substance, still more marked is the relief afforded when they can bite upon some hard, cold substance, which in addition to mechanically lessening the blood supply, causes contraction of the dilated vessels.

### PATHOLOGICAL FIRST DENTITION.

The local disturbances may be exaggerated beyond the degree accepted as physiological and may be accompanied by general nervousness, alimentary, pulmonary and cutaneous disturbances. This is pathological dentition and may be of several degrees of severity. The primary cause of pathological dentition may be stated as an inequality in the rate of gum absorption and tooth advance. The advancing crown of the tooth pressing on the gum tissue causes irritation, as Hyperemia or mild aseptic inflammation resulting, instead of remaining at a point favoring the development of giant cells and resorption, it passes the physiological point and causes a disturbance of function. Inflammation simple or infective may occur in the area. Swelling of the gum occurs, which, being distributed in all directions, presses on the crown of the tooth depressing it upon the pulp beneath the sharp root margins and at the same time the blood pressure of the pulp tends to press the tooth upward. The sharp edges of the root must irritate the pulp tissue, which is composed of connective tissue, nerves and blood vessels, which become inflamed and swollen, and still more strongly urges the tooth upward. Two sources of irritation are now possible. The irritated gum tissue and the irritated pulp. The pulp is more likely to cause reflex disturbance through the intimate sympathetic relations that exist between the fifth cranial nerve, which supplies the teeth, and the seventh, ninth and tenth in the floor of the fourth ventricle; so that salivary, muscular, alimentary and pulmonary disturbances become possible as the result of the referred pain produced by the irritation of the pulp of the erupting tooth. Though the pulp is more likely to produce reflex disturbance, an overlying gum inflammation is capable of producing even prostrating symptoms. Any systemic disturbance which interferes with the general nutritive functions also in the parts associated with the teeth may favor the production of local pathological conditions.

Pathological dentition may occur in the absence of evident hyperemic gum tissue, the tissue may be white, showing Ischemia from pressure, a binding down on the end of the root and upon the pulp—being proved by the subsidence of symptoms after lancing, and sometimes by the rapid partial eruption of the tooth after lancing. Again, pathological phenomena have been noted where no superficial disturbance was manifest. In these cases the deep tissues may exert restraining influence upon the crown, but the swelling is just as probable.

It is to be understood that the Nervous and Digestive systems of the child are in a developmental condition and, therefore, in unstable physiological equilibrium, so that any added physiological work, such as unusual growth or dentition, may be more than the organism can endure without a definite loss of general vital potentiality. This may be further complicated by hereditary defects of tissue; such as neurotic, degenerative, syphilitic taint or conditions of hygiene or feeding, tending to lower the health standard.

### SYMPTOMS.

The symptoms of pathological dentition are both local and general.

### LOCAL SYMPTOMS.

The local symptoms are usually those of inflammation, red and swollen gum tissue—at times these assuming a dusky hue. The gums may be white, often glistening, indicating their stretching over the crown.

In the gum overlying the erupting tooth there may exist a vascular enlargement containing fluid. Evidence of local irritability is evidenced by the fact that the child resists the touching of the gums, seizing the breast or bottle nipple and immediately releases it. The readiness with which the child will take in the mouth cold solids and ice water is self-explainable. Alternate excessive flow of saliva and oral dryness is present.

In more marked cases of local disturbance indicating bacterial infection of the mucous membrane of the mouth may appear, such as ulcerative stomatitis, as a rule this breaking down and ulceration is usually confined to the parts overlying the erupting teeth. A general stomatitis or widly scattered patches of ulceration may also make their appearance.

### GENERAL SYMPTOMS.

General symptoms may be differentiated into mild and severe. The mild symptoms are such as may be seen upon severe and painful inflammations about the face at almost any age, thus anorexia, fretfulness, anger, restlessness, sleeplessness, thirst, mild fever, and evident desire for the upright position occurs. The pain is at times paroxymal, but may be continuous. These symptoms disappear when the tooth erupts, or when the tissue overlying the tooth is lanced, however, if the adjoining teeth exert any restraint upon the erupting tooth, the symptoms may exist for a while even after these measures bave been taken. The more severe general symptoms are such as are brought about by reflex neurosis.

The root of the fifth cranial nerve supplying the teeth is in intimate relation with the roots of the seventh, ninth and tenth cranial nerves in the floor of the ventrical and it may be argued that upon these grounds that irritation of the peripheral ends of the fifth, in the pulp tissue of the tooth may therefore readily produce neurotic results in the brain, salivary glands, skin, lungs and larynx, intestinal canal or muscles of the face or extremities. Taking the intestinal canal as the most complicated example we find the following data: The stomach and the intestines are under the influence of the Pneumogastric nerve, which sends to its muscular coats both stimulant and inhibitory fibres, in like manner it sends vasomotor fibers to the intestines, divisions which lead to inhibition of the muscular fibres of the vessel and leads to vasodilation and a great increase of the intestinal juices.

That intestinal disturbances may arise independently of teething is self evident. They are more liable to occur during the period at which teething may be supposed to act as a primary cause of intestinal troubles, hence differentation becomes important. That the conditions may be associated is also evident. As a rule, intestinal disturbances arise from improper feeding, the food acting as an indigestible irritant to the stomach and intestines. Even a large quantity of breast milk or good bottle milk if not regurgitated acts as an intestinal irritant. The milk of an excited or debauched nurse may also act deleteriously.

Fermentation due to bacteria, diarrhea and colic are a natural result. These conditions are attributed to the development of the Baccillus Coli Communis and the Baccilli Lactis Aeriformis, existing harmlessly in the normal intestine, but developing excessively under abnormal conditions. This occurring in warm weather when the child suffers from intense heat may have a very debilitating, if not a fatal result. The condition may be viewed as an infective diarrhea following a vasomotor disturbance of the intestinal walls set up by reflexes primarily caused by indigestible food, the vital resistance being caused by the disturbed alimentation and pain.

A similar train of causes may be developed by teething. Peripheral irritation of the terminals of the fifth nerve in the pulp may cause through the pneumogastric nerve, a reflex vasomotor dilation in the walls of the intestines, a condition which favors bacterial invasion. Intestinal digestion is disordered, the vital resistance lowered and an infection ordinarily resisted occurs. Alimentation is interfered with and the general nutrition suffers. The child is debilitated by lack of nutrition, moreover, toxic substances are generated in the intestines which cause a toxemia to which many of the general symptoms may be attributed. such as fever, meningitis, stupor, coma, and death.

The general debility further interferes with the process of dentition. A diarrhea due to improper feeding would not be preceded by symptoms of pathological dentition, but would have a history of improper feeding and possibly of un-hygienic conditions, such as unsterilized milk, or dirty milk bottles and filthy surroundings. There is a catarrhal diarrhea accompanied by vomiting and constant acid watery stools. Such an infected diarrhea may readily follow the reflex and debilitating effects of pathological dentition, as stated before. White has noted that a choleric diarrhea may accompany and be a sign of pathological dentition. Barrett states that a diarrhea due to dentition will usually be followed by constipation. A symptomatic diarrhea will, as a rule, be accompanied by signs of a pathological dentition at points in the jaws at which teeth should be in the process of eruption.

### NERVOUS DISTURBANCES.

Disorders referable to the central nervous system are the most alarming and are those indicating the higher grades of severity of irritation. The milder forms of them are faint muscular twitchings and evidence of slight cerebral disturbance. A distressing symptom not easy to account for on account of the age of the patient is headache. The child is sleepless and cries without apparent cause, it becomes quiet partially from exaustion and after a period again commences sobbing. The indication of central nervous disturbance may, at times, be noted by the contracted conditon of the pupils of the eyes and in throbbing arteries.

In the severe and more dangerous cases the evidence of disorders of the central nervous system becomes unmistakable. These appear as chronic convulsions or symptomatic eclampsia. While it is probable in many cases that reflex irritation from the process of dentition in itself is but the secondary cause of convulsions, yet evidence is sufficient to warrant its being regarded as a determining factor.

In very many cases, teething convulsions appear to indicate neurotic family taint, and eclampsia may attend disorders in children of this type, notably the mechanical and chemical irritation induced by the presence of large masses of indigestible food in the intestines. So called teething convulsions usually accur at a time when several teeth are in the process of eruption, the onset of the convulsions is rarely, although apparently often, sudden. If the child be closely observed, it is noted that a period of cerebral disturbance, fretful crying, evidence of headache, sleeplessness, etc., is followed by a period of dullness or the child may lie with eyes half open. Twitching of one or more groups of muscles may be observed, the orbicularis oris and other muscles of the lips and the muscles of the eye may contract spasmodically. A common muscular spasm ushering in convulsion is that of the adductor muscles of the thumb, the thumbs being drawn towards the palm of the hands. The adductor muscles of the feet being contracted the feet are drawn inward. This period may be ushered in with a sharp cry, the eyes rolled upward with the lids half open and consciousness is lost. The symptoms may disappear, the child awaken dazed and fretful, or it may sink into sleep. Unless the source of irritation be removed or active therapeutic measures be instituted the eclampsia may return and in severe cases death result.

### CONSTITUTIONAL STATES MODIFYING DENTITION.

Children who are the victims of hereditary syphilis usually cut their teeth very early, the alveolar process being in many cases insufficient. Cases are recorded where children have been born with crowns of teeth visible on the gums, there being no evidence of root formation, the crowns being loosely held to the gums by fibrous tissue. It is necessary to remove these loose crowns to permit the child to get nourishment from the breast.

